# Evaluation of Nowcasting for Detecting and Predicting Local Influenza Epidemics, Sweden, 2009–2014

**DOI:** 10.3201/eid2410.171940

**Published:** 2018-10

**Authors:** Armin Spreco, Olle Eriksson, Örjan Dahlström, Benjamin John Cowling, Toomas Timpka

**Affiliations:** Linköping University, Linköping, Sweden (A. Spreco, O. Eriksson, Ö. Dahlström, T. Timpka);; Center for Health Services Development, Region Östergötland, Linköping (A. Spreco, T. Timpka); Hong Kong University, Hong Kong Special Administrative Region, China (B.J. Cowling);

**Keywords:** epidemiology, infectious disease, human influenza, signal detection analysis, surveillance, evaluation research, nowcasting, viruses, influenza, Sweden

## Abstract

The growing availability of big data in healthcare and public health opens possibilities for infectious disease control in local settings. We prospectively evaluated a method for integrated local detection and prediction (nowcasting) of influenza epidemics over 5 years, using the total population in Östergötland County, Sweden. We used routine health information system data on influenza-diagnosis cases and syndromic telenursing data for July 2009–June 2014 to evaluate epidemic detection, peak-timing prediction, and peak-intensity prediction. Detection performance was satisfactory throughout the period, except for the 2011–12 influenza A(H3N2) season, which followed a season with influenza B and pandemic influenza A(H1N1)pdm09 virus activity. Peak-timing prediction performance was satisfactory for the 4 influenza seasons but not the pandemic. Peak-intensity levels were correctly categorized for the pandemic and 2 of 4 influenza seasons. We recommend using versions of this method modified with regard to local use context for further evaluations using standard methods.

Although the seasonal variations in influenza incidence among nations and global regions are well described (*1*), the duration and intensity of influenza epidemics in local communities have been less well monitored and understood. The rapidly growing availability of big data from diagnostic and prediagnostic (syndromic) sources in healthcare and public health settings opens new possibilities for increasing the granularity in infectious disease control (*2*,*3*). However, development of outbreak models and efficient use of the information produced by prediction models in public health response decision-making remain challenging. This observation was recently highlighted by the Congress of the United States request that the Government Accountability Office gather information on validation of emerging infectious disease model predictions (https://energycommerce.house.gov/wp-content/uploads/2017/11/20171109GAO.pdf).

We previously reported the design of a nowcasting method (detection of influenza epidemics and short-term predictions) for local-level application in the northwestern region of the world (*4*). In other fields, such as meteorology, nowcasting methods represent standard tools for warning the public against dangerous high-impact events (*5*). The rationale for developing this novel method was to inform the planning of local responses and adjustments of healthcare capacities. Many such adjustments are planned and performed locally, at county and municipality levels. In Sweden, for instance, the hospital bed capacity is habitually overextended; on average, 103 patients occupy 100 regular hospital bed units (*6*). It is therefore important that an influenza epidemic is noticed early at the local level to make time for implementation of adjustments (e.g., freeing hospital beds by removing from the waiting list those patients scheduled for elective interventions).

 We performed a prospective 5-year evaluation of local influenza nowcasting by using routine health information system data. The evaluation period included 1 pandemic (2009) and 4 winter influenza seasons (Figure). The nowcasting method is based on mathematical modeling of epidemic curves generated from historic local data (*4*). Nowcasting comprises 3 functions: detection of the local start of the epidemic, prediction of peak timing, and prediction of peak intensity.

**Figure:**
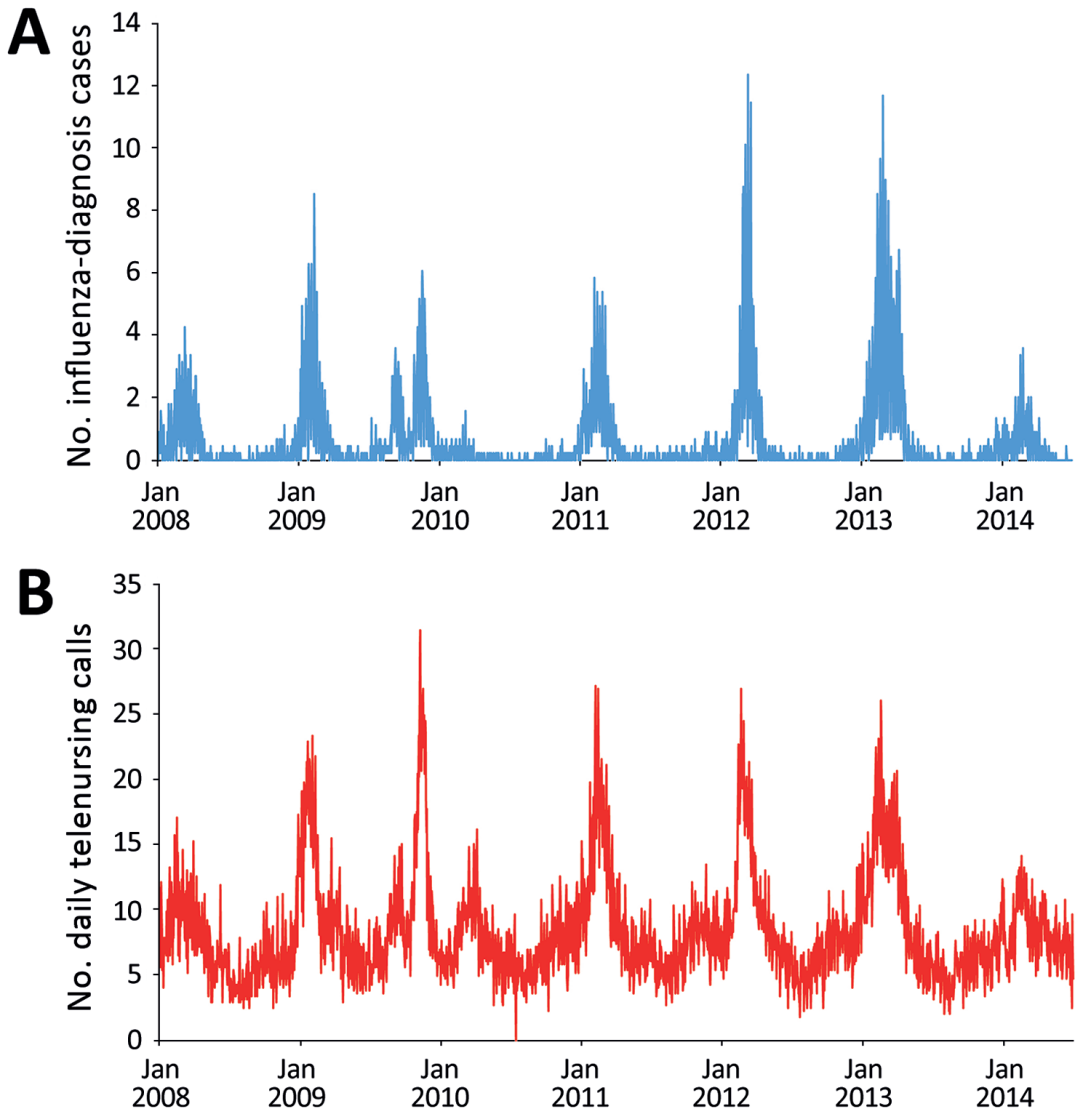
Data used for evaluation of nowcasting for detection and prediction of local influenza epidemics, Östergötland County, Sweden, January 1, 2008, through June 30, 2014. A) Unadjusted daily numbers of influenza-diagnosis cases per 100,000 population. B) Unadjusted daily telenursing calls because of fever (child and adult) per 100,000 population.

## Methods

We used an open cohort design based on the total population (n = 445,000) in Östergötland County, Sweden. We used prospective data from July 1, 2009, through June 30, 2014, from 2 sources in the countywide health information system: clinical influenza-diagnosis cases recorded by physicians and syndromic chief complaint data from a telenursing service (*4*). The influenza-diagnosis case data were used for detection of the local start of the epidemic and prediction of its peak intensity; the syndromic data were used for prediction of the peak timing. Timeliness was used as a performance metric for detection of the local start of the epidemic and the peak-timing prediction; the correct identification of intensity category on a 5-grade scale was used for peak-intensity prediction. The study design was approved by the Regional Research Ethics Board in Linköping (no. 2012/104–31).

### Definitions

We identified influenza cases by using codes from the International Classification of Diseases, 10th Revision, for influenza (J10.0, J10.1, J10.8, J11.0, J11.1, J11.8) (*7*) as recorded in the local electronic health data repository. We identified influenza-related telenursing call cases by using the chief complaint codes associated with influenza symptoms (dyspnea, fever [child and adult], cough [child and adult], sore throat, lethargy, syncope, dizziness, and headache [child and adult]).

The intensity level for the start of a local epidemic (i.e., the endpoint for the detection function) was set to 6.3 influenza-diagnosis cases/100,000 population recorded during a floating 7-day period in the countywide health information system (*3*). A recent comparison of influenza intensity levels in Europe estimated a similar definition (6.4 influenza-diagnosis cases/wk/100,000 population) for the 2008–09 winter influenza season in Sweden (*8*). Peak timing was defined as the date when the highest number of influenza-diagnosis cases were documented in the countywide electronic patient record. Peak intensity was defined as the number of influenza-diagnosis cases that had been documented on that date.

The detection threshold was adjusted to situations when extended simmering of influenza-related activity appears before an epidemic. Such simmering was associated with exceptionally mild winter influenza seasons and pandemics. Preepidemic simmering before winter influenza seasons was defined as occurring when the period between increased influenza incidence above baseline and the start of the epidemic is prolonged (*4*). The upper threshold for the baseline was set to 3.2 influenza-diagnosis cases/100,000 population during a floating 7-day period (i.e., half of the defined start-of-epidemic intensity level). If the baseline threshold was surpassed for a period 3 times longer than the average period before previous epidemics without exceeding the start-of-epidemic level (i.e., 6.3 influenza-diagnosis cases/100,000 population during a floating 7-day period), preepidemic simmering was considered to have occurred. Preepidemic simmering in association with a pandemic was defined as that ensuing from the date of a World Health Organization pandemic alert. We determined all definitions and adjustments before using the method for detection and prediction.

### Method Application

Technical details concerning the 3 functions of nowcasting are provided in Technical Appendix 1. The programming code for the analyses is provided in Technical Appendix 2.

To calibrate the detection component of the nowcasting method, we retrospectively determined weekday effects on recording of influenza-diagnosis cases and a baseline alarm threshold by using learning data. These data were collected from January 1, 2008, through June 30, 2009, including the 2 winter influenza seasons 2007–08 and 2008–09. To determine weekday effects, we used data from the entire learning data collection period. To determine the initial alerting threshold, we used only data from the winter influenza season in 2008–09. The 2007–08 winter influenza season could not be used for this purpose because collection of learning data had already started. Throughout the study period, the calibration data were updated after every winter influenza season (i.e., no updates after the 2009 pandemic outbreak). The detection algorithm was thus applied to the next epidemic by using the revised threshold determined in the updated learning dataset. We identified 2 exceptional situations: pandemic settings and winter influenza seasons that simmered before they started (*4*). In these situations, the alarm threshold is doubled. Accordingly, following the World Health Organization pandemic alert (*9*), the alarm threshold was doubled for the 2009 season. Before the 2010–11 winter influenza season, the threshold was reset to the regular level. No updates were performed because the set of learning data remained the same (i.e., it contained data from the 2008–09 winter influenza season). For the 2011–12 winter influenza season, we updated the threshold by using learning data from the 2008–09 and 2010–11 winter influenza seasons. For the 2012–13 winter influenza season, we updated the threshold by using learning data from the 2008–09, 2010–11, and 2011–12 winter influenza seasons. For the 2013–14 winter influenza season, we again updated the alerting threshold by using learning data from the previous winter influenza seasons (2008–09, 2010–11, 2011–12, and 2012–13). However, because this winter influenza season simmered before it started, the threshold was doubled before the detection method was applied. The weekday effects were assumed to be relatively constant over time in the local detection analyses and therefore were not updated after every winter influenza season.

We also used the set of learning data from the winter influenza seasons in 2007–08 and 2008–09 to initially calibrate the first component of the local prediction module. The dataset was used to decide the grouping of chief complaints with the largest correlation strength and longest lead time between influenza-diagnosis data and telenursing data (*10*,*11*). The best performing telenursing chief complaint was fever (child and adult), and the most favorable lead time was 14 days. When the peak timing had been determined, the second component of the local prediction module was applied to influenza-diagnosis data from the corresponding epidemics to find the peak intensity on the predicted peak day (*4*). Regarding weekday effects on local prediction, the same calculation was applied and the same grouping of chief complaints and lead time were used throughout the study.

### Metrics and Interpretations

For trustworthiness of the nowcasting method in local healthcare planning, we set the maximum acceptable timeliness error for detection and peak timing predictions to 1.5 weeks. Method performance was defined to be excellent if the absolute value of the timeliness error was <3 days, good if it was 4–7 days, acceptable if it was 8–11 days, and poor if it was >12 days. For peak intensity predictions, we used the epidemic threshold and intensity level categories (nonepidemic, low, medium, high, and very high) identified for Sweden in a study involving 28 European countries (*8*) (Table 1). If the predicted peak intensity fell into the same category as the actual peak intensity, the prediction was considered successful; otherwise, it was considered unsuccessful.

**Table 1:** Epidemic intensity categories used to interpret performance measurements in evaluation of nowcasting for detection and prediction of local influenza epidemics, Sweden, 2009–2014*

Intensity level	Threshold, cases/d/100,000 population					
2008–09	2009 pandemic	2010–11	2011–12	2012–13	2013–14
Nonepidemic	<0.9	<0.9	<0.9	<1.0	<1.2	<1.2
Low	0.9	0.9	0.9	1.0	1.2	1.2
Medium	2.4	2.5	2.5	2.5	2.8	2.9
High	5.5	5.4	5.4	5.2	5.6	5.5
Very high	7.9	7.5	7.5	7.1	7.7	7.4

*Based on (*8*).

## Results

### Local Detection

The detection component of the local nowcasting method showed good performance during the 2009 pandemic of influenza A(H1N1)pdm09 (pH1N1) virus (Table 2), alerting for the local influenza epidemic 5 days after it actually started. For the 2010–11 winter influenza season with influenza B and pH1N1 viruses circulating, the local detection performance was also good; the alarm was raised 5 days after the start of the local epidemic. During the 2011–12 winter influenza season, with influenza A(H3N2) virus activity only, detection performance was poor. During that season, the alarm was raised 15 days before the actual local epidemic started. During the 2012–13 and 2013–14 winter influenza seasons, with influenza A(H3N2), influenza B, and pH1N1 viruses circulating, the local detection performance was excellent; alarms were raised 3 days before the 2012–13 epidemic started and 3 days after the 2013–14 epidemic started.

**Table 2:** Performance of the detection algorithm displayed with alert thresholds updated by using data from previous winter influenza seasons in evaluation of nowcasting for detection and prediction of local influenza epidemics, Sweden, 2009–2014*

Influenza virus activity	Updated threshold, cases/d/100,000 population	Timeliness†	Interpretation
2009 pH1N1‡	0.424	−5	Good
2010–11 B and pH1N1	0.212	−5	Good
2011–12 A(H3N2)	0.207	15	Poor
2012–13 A(H3N2), B, and pH1N1	0.242	3	Excellent
2013–14 A(H3N2), B, and pH1N1‡	0.481	−3	Excellent

*pH1N1, pandemic influenza A(H1N1)pdm09 virus. †Positive value means that the algorithm issued an alarm before the local epidemic had started; negative value means that the alarm was raised after the start of the epidemic. ‡The threshold was doubled because of a pandemic alert or observation of a period of simmering influenza activity.

### Local Prediction

For the 2009 influenza pandemic, performance of the local peak-timing prediction was poor, but the peak-intensity level was correctly categorized (medium intensity epidemic) (Table 3). For the 2010–11 winter influenza season, with influenza B and pH1N1 viruses circulating, the local peak-timing prediction was excellent and the peak intensity was successfully predicted to the correct category (medium intensity). For the 2011–12 winter influenza seasons with influenza A(H3N2) virus circulating and the 2012–13 winter season with influenza A(H3N2), influenza B, and pH1N1 viruses circulating, the local peak-timing predictions were good. The local peak-intensity predictions were successful for the 2012–13 winter influenza season, correctly categorizing it to a very high intensity level and unsuccessful for the 2011–12 season, categorizing it as a medium intensity epidemic when it actually developed into a very high-intensity epidemic. For the 2013–14 winter influenza season, with influenza B and pH1N1 viruses circulating and a simmering start, the local peak-timing prediction was acceptable, but the local peak intensity was wrongly predicted to be at the nonepidemic level when the winter influenza season actually reached a medium intensity level.

**Table 3:** Performance of peak-timing and peak-intensity predictions from evaluation of nowcasting for detection and prediction of local influenza epidemics, Sweden, 2009–2014*

Influenza virus active	Time-of-peak predictions†					Peak intensity predictions		
Date when prediction made	Time to peak, d	Prediction error	Interpretation	Category (cases/d/100,000 population)		Interpretation
				Predicted	Factual	
2009 pH1N1	2009 Sep 27	8	−28	Poor		Medium (3.3)	Medium (2.9)	Successful
2010–11 B and pH1N1	2011 Feb 11	10	0	Excellent		Medium (4.5)	Medium (4.9)	Successful
2011–12 A(H3N2)	2012 Feb 25	9	7	Good		Medium (4.5)	Very high (12.4)	Unsuccessful
2012–13 A(H3N2), B, and pH1N1	2013 Feb 22	10	−7	Good		Very high (10.1)	Very high (11.7)	Successful
2013–14 A(H3N2), B, and pH1N1	2014 Feb 17	8	−8	Acceptable		Nonepidemic (1.0)	Medium (3.4)	Unsuccessful

*pH1N1, pandemic influenza A(H1N1)pdm09 virus. †Positive value means that the peak was predicted to be reached before the actual peak occurred; negative value means that the peak was predicted to be reached after the actual peak occurred.

## Discussion

In this prospective 5-year evaluation of a method for local nowcasting of influenza epidemics that used routine health information system data, we identified aspects that were satisfactory and identified areas where improvements are needed. The detection function displayed satisfactory performance throughout the evaluation period, except for the 2011–12 winter influenza season, in which influenza A(H3N2) virus circulated after a season with influenza B and pH1N1 virus activity. Peak-timing prediction performance was satisfactory for the 4 winter influenza seasons but not for the 2009 pandemic. In addition, the method categorized the local peak-intensity levels correctly for the 2009 pandemic and for 2 of the winter influenza seasons, but it was unsuccessful at forecasting the very high peak intensity of the 2011–12 season and the medium peak intensity of the 2013–14 season, which was preceded by a simmering phase.

The results indicate that securing the availability of a new data source is only the first step toward using the data stream in routine surveillance. The syndromic data source used for the big data stream in this study was subjected to rigorous restructuring and maintenance. Nonetheless, not all parameters associated with the syndromic data stream were regularly updated. For the peak-timing predictions made by using telenursing data, we assumed that increases in telenursing activity precede influenza diagnoses by 14 days. Although this assumption is grounded (*10*,*11*), the interval may change over time and thereby influence influenza predictions. Using the constant interval estimate, we estimated the influenza diagnosis peaks for the 2011–12 and 2012–13 winter influenza seasons 1 week before and 1 week after the actual influenza-diagnosis peaks. In other words, the basic prediction method may have been more accurate at predicting the peak timing than the results of this study show. In the setting of our study, the performance was most likely decreased by the assumption that telenursing precedes influenza diagnosis by 14 days is applicable to all situations. Althouse et al. reported that methods underpinning the use of big data sources (e.g., search query logs) need regular upkeep to maintain their accuracy (*12*). In future versions of nowcasting methods, regular updates and syndromic sources that are more stable than telenursing data (regarding time lag to influenza diagnosis data) may become available and can be used to improve the peak-timing predictions.

Influenza forecasting is methodologically challenging (*13*,*14*), and only a few prospective evaluations have transparently reported algorithms and study designs. In the first Centers for Disease Control and Prevention (CDC) challenge, a prospective study of state-of-the-art methods in which 4 aspects of influenza epidemics (start week, peak week, peak percentage, and duration) were forecasted by using routine data (*15*), none of the evaluated methods showed satisfactory performance for all aspects. Similarly, in the second CDC challenge, in which 3 aspects of influenza epidemics (start week, peak week, and peak intensity) were forecasted by using 7 methods, none of the evaluated methods displayed satisfactory performance (*16*). These challenge studies have substantially helped to widen the understanding of the difficulties of forecasting different aspects of influenza epidemics. In our study, the detection and peak-intensity prediction functions of the local nowcasting method underperformed during the 2011–12 winter influenza season. One reason for the observed underperformance may be that the data from preceding seasons used to generate local epidemic curves were insufficient for modeling the between-seasons drift in the immunity status of the population in relation to the circulating influenza strain. In other words, the present parameters used to compute epidemic curves were deficient when large drifts in population immunity with corresponding changes in virus dissemination patterns occurred. For instance, the epidemic phase of the influenza A(H3N2) season in 2011–12 may not have started with virus spread among the young persons in the community as it had during previous seasons (*17*,*18*). It has been suggested that including virologic information (i.e., influenza virus type and subtype) as model parameters may improve the predictive accuracy of mathematical models (*19*). We contend also that, for local influenza detection and prediction, historical accounts of the circulating influenza virus types should be considered for inclusion in the statistical models and suggest adding information about the population age structure. However, such model extensions must also be paralleled by securing a continuous supply of the corresponding data in the local settings where the models are to be used.

This study has strengths and weaknesses that need to be considered when interpreting the results. The main strength of the study is that it prospectively evaluates an integrated influenza nowcasting method in a local community. On the basis of experiences from previous studies (*4*,*15*,*16*,*20*), we considered timeliness to be the most valid general evaluation metric for our purposes. To be able to accurately support adjustments of local healthcare capacity, we used daily data for the evaluations. In the CDC challenge studies (*15*,*16*), forecasts of the start and peak timing of an epidemic were based on weekly data and considered accurate if they occurred within 1 week of the actual timing of each component. Therefore, we consider the limits used for evaluating detection and the peak-timing predictions in this study to be at least as strict as those in the CDC challenge. Regarding the prediction of peak intensity, we considered a forecast to be accurate if it predicted the peak intensity to be the correct peak-intensity category as defined by Vega et al. (*8*), who calculated the thresholds for each of these categories for every winter influenza season by applying the moving epidemic method (*21*) on 5–10 previously occurring seasons. Hence, we consider these categories to be reliable. A longer prospective evaluation period would have increased the possibility of drawing valid conclusions concerning the outcome of the evaluation, and it would be preferable to have corresponding local data from other cities or regions (*22*). Evaluating our nowcasting method for epidemics from several other regions would enable conclusions to be drawn about the generalizability of the method.

We contend that methods for local nowcasting of influenza epidemics based on routine health information system data have potential for general dissemination and use. Future versions of the nowcasting model will be gradually extended with information on population age distribution and on current and previously circulating influenza types. Such extensions need to be paralleled by securing a routine supply of data to the added parameters in local health information systems. We recommend using versions of the nowcasting method modified with regard to their local use context for further evaluations with standard measures.

**Technical Appendix 1.** Method design overview of evaluation of nowcasting for detection and prediction of local influenza epidemics, Sweden, 2009–2014.

**Technical Appendix 2.** Programming code used in evaluation of nowcasting for detection and prediction of local influenza epidemics, Sweden, 2009–2014.

## References

[1] Polansky LS, Outin-Blenman S, Moen AC. Improved global capacity for influenza surveillance. Emerg Infect Dis. 2016;22:993–1001. 10.3201/eid2206.151521 27192395 PMC4880096

[2] Shaman J, Karspeck A, Yang W, Tamerius J, Lipsitch M. Real-time influenza forecasts during the 2012-2013 season. Nat Commun. 2013;4:2837. 10.1038/ncomms3837 24302074 PMC3873365

[3] Riley RD, Ensor J, Snell KI, Debray TP, Altman DG, Moons KG, et al. External validation of clinical prediction models using big datasets from e-health records or IPD meta-analysis: opportunities and challenges. BMJ. 2016;353:i3140. 10.1136/bmj.i3140 27334381 PMC4916924

[4] Spreco A, Eriksson O, Dahlström Ö, Cowling BJ, Timpka T. Integrated detection and prediction of influenza activity for real-time surveillance: algorithm design. J Med Internet Res. 2017;19:e211. 10.2196/jmir.7101 28619700 PMC5491899

[5] Bližňák V, Sokol Z, Zacharov P. Nowcasting of deep convective clouds and heavy precipitation: comparison study between NWP model simulation and extrapolation. Atmos Res. 2017;184:24–34. 10.1016/j.atmosres.2016.10.003

[6] Sveriges Kommuner och Landsting. Ingen på sjukhus i onödan. No one in hospital unnecessarily [in Swedish]. Stockholm: Sveriges Kommuner och Landsting; 2016.

[7] World Health Organization. International statistical classification of diseases and related health problems. 10th Revision, vol. 2. Geneva: The Organization; 2010.

[8] Vega T, Lozano JE, Meerhoff T, Snacken R, Beauté J, Jorgensen P, et al. Influenza surveillance in Europe: comparing intensity levels calculated using the moving epidemic method. Influenza Other Respi Viruses. 2015;9:234–46. 10.1111/irv.12330 26031655 PMC4548993

[9] World Health Organization. Influenza A(H1N1) [cited 2017 Jan 15]. http://www.who.int/mediacentre/news/statements/2009/h1n1_20090429/en/

[10] Timpka T, Spreco A, Dahlström Ö, Eriksson O, Gursky E, Ekberg J, et al. Performance of eHealth data sources in local influenza surveillance: a 5-year open cohort study. J Med Internet Res. 2014;16:e116. 10.2196/jmir.3099 24776527 PMC4019774

[11] Timpka T, Spreco A, Eriksson O, Dahlström Ö, Gursky EA, Strömgren M, et al. Predictive performance of telenursing complaints in influenza surveillance: a prospective cohort study in Sweden. Euro Surveill. 2014;19:46. 10.2807/1560-7917.ES2014.19.46.20966 25425514

[12] Althouse BM, Scarpino SV, Meyers LA, Ayers JW, Bargsten M, Baumbach J, et al. Enhancing disease surveillance with novel data streams: challenges and opportunities. EPJ Data Sci. 2015;4:17. 10.1140/epjds/s13688-015-0054-0 27990325 PMC5156315

[13] Chretien JP, George D, Shaman J, Chitale RA, McKenzie FE. Influenza forecasting in human populations: a scoping review. PLoS One. 2014;9:e94130. 10.1371/journal.pone.0094130 24714027 PMC3979760

[14] Lazer D, Kennedy R, King G, Vespignani A. Big data. The parable of Google Flu: traps in big data analysis. Science. 2014;343:1203–5. 10.1126/science.1248506 24626916

[15] Biggerstaff M, Alper D, Dredze M, Fox S, Fung IC, Hickmann KS, et al.; Influenza Forecasting Contest Working Group. Results from the centers for disease control and prevention’s predict the 2013-2014 Influenza Season Challenge. BMC Infect Dis. 2016;16:357. 10.1186/s12879-016-1669-x 27449080 PMC4957319

[16] Biggerstaff M, Johansson M, Alper D, Brooks LC, Chakraborty P, Farrow DC, et al. Results from the second year of a collaborative effort to forecast influenza seasons in the United States. Epidemics. 2018 Feb 24;pii:S1755–4365(17)30088–9. Epub ahead of print [cited 2018 Jun 14]. http://dx, 10.1016/j.epidem.2018.02.003 PMC6108951 29506911

[17] Schanzer D, Vachon J, Pelletier L. Age-specific differences in influenza A epidemic curves: do children drive the spread of influenza epidemics? Am J Epidemiol. 2011;174:109–17. 10.1093/aje/kwr037 21602300 PMC3119537

[18] Timpka T, Eriksson O, Spreco A, Gursky EA, Strömgren M, Holm E, et al. Age as a determinant for dissemination of seasonal and pandemic influenza: an open cohort study of influenza outbreaks in Östergötland County, Sweden. PLoS One. 2012;7:e31746. 10.1371/journal.pone.0031746 22384066 PMC3285651

[19] Moniz L, Buczak AL, Baugher B, Guven E, Chretien JP. Predicting influenza with dynamical methods. BMC Med Inform Decis Mak. 2016;16:134. 10.1186/s12911-016-0371-7 27756371 PMC5070096

[20] Cowling BJ, Wong IO, Ho LM, Riley S, Leung GM. Methods for monitoring influenza surveillance data. Int J Epidemiol. 2006;35:1314–21. 10.1093/ije/dyl162 16926216

[21] Vega T, Lozano JE, Meerhoff T, Snacken R, Mott J, Ortiz de Lejarazu R, et al. Influenza surveillance in Europe: establishing epidemic thresholds by the moving epidemic method. Influenza Other Respi Viruses. 2013;7:546–58. 10.1111/j.1750-2659.2012.00422.x 22897919 PMC5855152

[22] Moss R, Zarebski A, Dawson P, McCAW JM. Retrospective forecasting of the 2010-2014 Melbourne influenza seasons using multiple surveillance systems. Epidemiol Infect. 2017;145:156–69. 10.1017/S0950268816002053 27671159 PMC9507331

