# Supplementary material for: Evaluation of Nowcasting for Detecting and Predicting Local Influenza Epidemics, Sweden, 2009–2014
Source: Emerg Infect Dis. 2018 Oct;24(10):1868–73. doi: 10.3201/eid2410.171940 (PMC6154154; doi:10.3201/eid2410.171940)
Supplement: Supplementary file 1 — Technical Appendix 1. Method design overview of evaluation of nowcasting for detection and prediction of local influenza epidemics, Sweden, 2009–2014. [file 17-1940-Techapp-s1.pdf]

Article DOI: https://doi.org/10.3201/eid2410.171940

Evaluation of Nowcasting for Detecting and Predicting Local Influenza Epidemics, Sweden, 2009–2014

Technical Appendix 1

Method Design Overview

The nowcasting method is divided into separate modules for detection and prediction of influenza activity, respectively (*1*). An overview of the main statistical assumptions and equations for each method component is displayed in the Technical Appendix Figure. The function of the detection module is to alert for an upcoming period of increased load of influenza-diagnosis cases on local health care services, whereas the function of the prediction module is to predict the timing of the activity peak and its intensity. The prediction process is divided into two components. In the first component, syndromic data are used to predict the peak timing, and in the second component, influenza-diagnosis data are used to estimate the peak intensity.

In the study setting, patients clinically diagnosed with influenza were used as gold standard. Early detection of increased influenza activity and prediction of peak intensity were thus based on streams of the gold standard data, whereas prediction of peak timing was based on syndromic data.

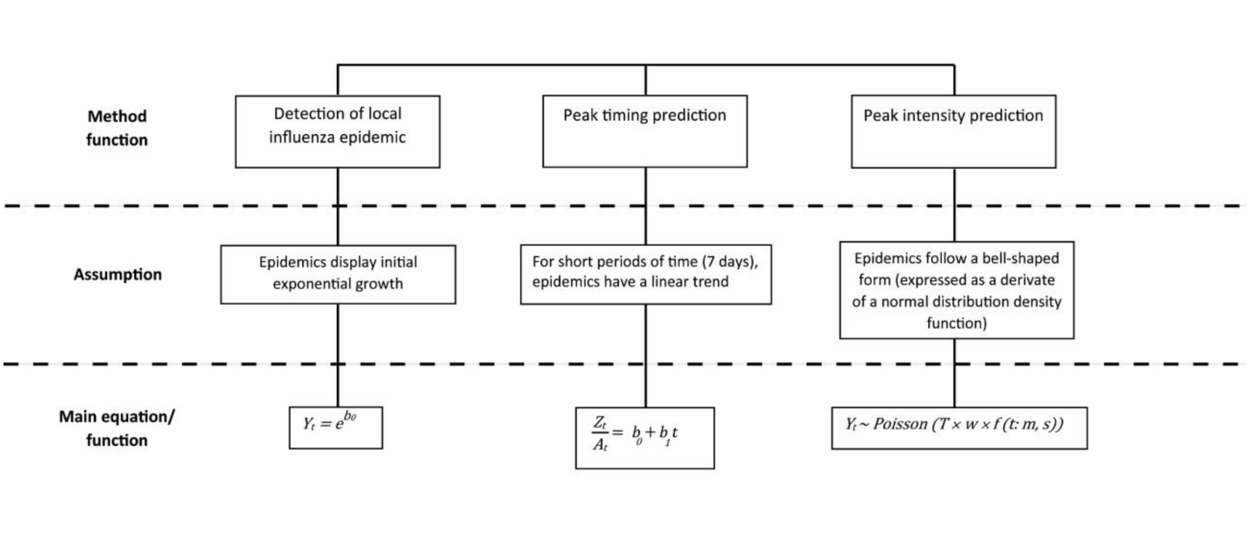

Figure. Overview of the main mathematical equations or functions used for each component.

Detection Module

Exponential regression (1) is used for detection modeling, based on the observation that the beginning of an influenza epidemic is assumed to have an exponential growth of infected individuals;

(1) $X_t = e^{a_0 + b_1 t}$,

with t representing the time, a_0 representing the level, and b_1 representing the trend. The expected number of visits at local health care services, $E[Y_t]$ is the product of X and the probability p for an infected individual to visit the local health care service. This expectation is also exponential in time;

(2) $E[Y_t] = e^{a_0 + b_1 t} p = e^{a_0 + ln(p) + b_1 t} = e^{b_0 + b_1 t}$,

Where b_0 now combines the current level of number of infected and probability of visiting the local health care service without any possibility to separate them. As daily data are used in the analysis, weekday effects, A_w, are also calculated and used as an offset variable in the exponential regression analysis. The weekday effects are calculated as follows: let A_{Monday} be the average number of events on Mondays during previous epidemics and denote the values for other weekdays by $A_{Tuesday}$, $A_{Wednesday,}$ and so on. Let $A_{Total} = (A_{Monday}+...+A_{Sunday})/7$. The multiplicative

weekday effect for Mondays is A_{Monday}/A_{Total} and so on. The weekday effects are included in the model;

(3) $E[Y_t] = e^{b_0 + b_1 t + ln(A_w)}$,

If X is large, p is small, and the infected individuals act independently, then Y is approximately Poisson distributed;

(4) $Y_t \sim Poisson\ (e^{b_0 + b_1 t + ln(A_w)})$.

Furthermore, the time is shifted, that is, the most recent day is considered as $t = 0$, the second most recent day is considered as $t = -1$, and so on. For every new day, the time axis is moved one step so that the new "most recent day" is considered as $t = 0$. For each day an exponential regression analysis (1) is run and a fitted value $\hat{y}$ is calculated by inserting $t = 0$ in equation (3) giving

(5) $Y_t = e^{b_0}$,

as an estimate of the current level of visits which is smoothed for random variation and adjusted for weekday effects. This is repeated for each day by moving the time axis 1 day at a time so that the most recent point in time of the series is considered $t = 0$. Doing this, one value is obtained for every day representing the level for that day. Finally, the lower 95% confidence limit is calculated to represent the level of influenza activity, which is then compared with a predetermined threshold. If the level (i.e., the lower 95% confidence limit) is above the threshold, an alarm is raised, which means that the winter influenza season (or pandemic) has started; and if the level is below the threshold, no alarm is raised.

Detection starts when the previous epidemic has ended (the interepidemic period level for the community where the detection component is applied), and runs during the inter-epidemic period until an increase in diagnosed influenza cases is detected. When the increase is confirmed, the algorithm is paused and restarted when the epidemic has ended.

Prediction Module

Peak Timing Prediction

In the first component, the aim is to predict the peak timing using linear regression. Including weekday effects A_w and smoothed for random variation, the model for the number of cases in syndromic data are expressed as

(6) $Z_t = (b_0 + b_1 t) \times A_w$,

with b_0 representing the level and b_1 representing the trend. Since the weekday effects A_w are known, a model smoothed for weekday effects and random variation can be expressed as

(7) $Z_t / A_w = b_0 + b_1 t$

For each 7-day period, a linear regression (7) is run and parameter estimates b_0 and b_1 are fitted. The idea is to estimate the trend in syndromic data for every 7-day period (the first period being days 1–7 and the second being days 2–8), from the beginning of an epidemic and until the peak is found. Although it is unlikely that an epidemic curve increases and decreases linearly, the assumption can be made that the trend during a short period of 7 days has almost a linear increase or decrease.

The search for the peak starts when the detection algorithm signals that an epidemic has taken off and continues until the peak is detected. To identify the peak timing, two conditions are set. As per the first condition, it is essential to ensure that the epidemic has a sufficiently sharp upward trend. The trend is therefore defined as sufficiently sharp when significantly positive ($p < 0.05$) trends b_1 have occurred either during two consecutive or during three different 7-day periods. When one of these events has occurred, the second condition is applied. According to this condition, when the first significantly negative trend (b_1) during a 7-day period has occurred, it is assumed that the peak has been reached on the first day of this period. However, there is a possibility that this 7-day period “overlaps” with a previous 7-day period, which includes a significantly positive trend. In that case, the first 7-day period with a significantly negative trend is ignored and the peak is instead assumed to appear during the second 7-day period with a significantly negative trend. The search is aborted if the peak is not found when the epidemic has already descended in the local setting where the algorithm is applied.

When the peak is found in the syndromic data, the 14 days preceding influenza-diagnosis data (*2*) is used to find the peak (in influenza-diagnosis data). In other words, if the peak in the syndromic data appears on day 0, the influenza-diagnosis peak is assumed to appear on day 14. However, it is possible that the peak in the syndromic data occurs on a day during the weekend but highly unlikely that the peak in influenza-diagnosis data occurs on one of these days as, for instance, primary care centers are closed during weekends in Sweden. Instead, it is reasonable to assume that the influenza-diagnosis peak occurs at the beginning of the week because individuals who suffer influenza symptoms during the weekend visit primary care centers when they reopen on Monday or possibly Tuesday. Adjustments are therefore made by moving the influenza-diagnosis peak to the following Monday if it is expected to occur on a Friday, Saturday, or Sunday according to syndromic data and to the previous Tuesday if the peak is expected to take place on a Wednesday or Thursday. If the peak is expected to occur on a Monday or Tuesday, no adjustments are made. In other words, in the first case the syndromic data precedes influenza-diagnosis data between 15 and 17 days, in the second case between 12 and 13 days, and in the third case 14 days.

Depending on what day of the week the peak in the syndromic data are expected to take place, the prediction of the influenza-diagnosis peak is made between 6 and 11 days before it is expected to occur, as the syndromic peak can be determined first after 6 days has passed of the syndromic data series.

Peak Intensity Prediction

In the second component of the prediction module, the aim is to predict only the peak intensity. Based on empirical assessments of previous epidemics, an epidemic adjusted for weekday effects is assumed to show a bell-shaped form from the beginning to the end, and can therefore be expressed using a derivate of a normal distribution density function. The intensity function must also include weekday effects and total number of events during the whole epidemic. Assuming that the peak timing is known (estimated in the first prediction component) and that an epidemic follows the bell-shaped function around the peak, the intensity function can be used to predict the peak intensity at time m.

Assume that day number $t = 1, 2, 3, \ldots, t_i$; the observed number of influenza-diagnosis cases is $y = y_1, y_2, y_3, \ldots, y_i$, and that

(8) $Y_t \sim Poisson\ (T \times w \times f\ (t{:}\ m, s)),$

where *T* is the total number of health care visits of the whole epidemic, *w* is the weekday effects, *f* is the normal distribution density function, *t* is the day number, *m* is the center of the epidemic (which coincides with *t* for the peak), and *s* is the spread in time. Since *t*, *w*, and *m* are known, only the parameters *T* and *s* are estimated using *y* in such way so that the likelihood is maximized. However, to do that, first appropriate starting values for these parameters need to be selected. Finally, using the known parameter *m* and the estimated parameters *T* and *s*, the peak intensity at time *m* is calculated by replacing *t* with *m* in equation (8).

It is important that the start of the series seems appropriate because the second prediction component assumes that the level is zero or at an interepidemic level at the start and it is not optimal that there are single or occasional spikes at the beginning of the series. For that reason, the start of the series should be a couple of weeks before an epidemic is detected.

References

1. Spreco A, Eriksson O, Dahlström Ö, Cowling BJ, Timpka T. Integrated detection and prediction of influenza activity for real-time surveillance: Algorithm design. J Med Internet Res. 2017;19:e211. PubMed http://dx.doi.org/10.2196/jmir.7101
2. Timpka T, Spreco A, Dahlström Ö, Eriksson O, Gursky E, Ekberg J, et al. Performance of eHealth data sources in local influenza surveillance: a 5-year open cohort study. J Med Internet Res. 2014;16:e116. PubMed http://dx.doi.org/10.2196/jmir.3099
