# Supplementary material for: Evaluation of Nowcasting for Detecting and Predicting Local Influenza Epidemics, Sweden, 2009–2014
Source: Emerg Infect Dis. 2018 Oct;24(10):1868–73. doi: 10.3201/eid2410.171940 (PMC6154154; doi:10.3201/eid2410.171940)
Supplement: Supplementary file 2 — Technical Appendix 2. Programming code used in evaluation of nowcasting for detection and prediction of local influenza epidemics, Sweden, 2009–2014. [file 17-1940-Techapp-s2.pdf]

Article DOI: https://doi.org/10.3201/eid2410.171940

Evaluation of Nowcasting for Detection and Prediction of Local Influenza Epidemics, Sweden, 2009–2014

Technical Appendix 2

Programming code

Detection Module

The analyses and coding of this module were performed in IBM SPSS Statistics. The code was written using the SPSS Syntax. Descriptive text/comment of the code is added by beginning a line using an asterisk (i.e., *) and is terminated by a period.

* Program:

* 1. Paste the data/time series into IBM SPSS Statistics, including the variables influenza-like-illness cases (ILI), time and logaritmic values of the weekday effects.

* 2. The dependent variable is the ILI cases.

* 3. The independent variable is the time, where time is “shifted,” i.e., the last point in time is equal to 0, the second last point in time is –1 and so on.

* 4. The logarithmic values of the weekday effects are used as an offset variable and these are calculated using the initial learning data.

* 5. Run the initial program below.

```
GENLIN ILI WITH Time

/MODEL Time INTERCEPT = YES OFFSET = Weekdayeffect

DISTRIBUTION = POISSON LINK = LOG

/CRITERIA METHOD = FISHER (1) SCALE = 1 COVB = MODEL
MAXITERATIONS = 100 MAXSTEPHALVING = 5 PCONVERGE = 1E-006(ABSOLUTE)
```

SINGULAR = 1E-012 ANALYSISTYPE = 3(WALD) CILEVEL = 95 CITYPE = WALD LIKELIHOOD = FULL

/MISSING CLASSMISSING = EXCLUDE

/PRINT SOLUTION.

* 6. Run the program below t-1 times, where t is the length of the time series (in days).

DEFINE !SimReg (NumLoops = !CMDEND)

!DO !i = 1 !TO !NumLoops

USE ALL.

COMPUTE filter_$ = (Time<0).

VARIABLE LABELS filter_$ 'Time<0 (FILTER)'.

VALUE LABELS filter_$ 0 'Not Selected' 1 'Selected'.

FORMATS filter_$ (f1.0).

FILTER BY filter_$.

EXECUTE.

COMPUTE Time = Time+1.

EXECUTE.

*Generalized Linear Models.

GENLIN ILI WITH Time

/MODEL Time INTERCEPT = YES OFFSET = Weekdayeffect

DISTRIBUTION = POISSON LINK = LOG

/CRITERIA METHOD = FISHER (*1*) SCALE = 1 COVB = MODEL MAXITERATIONS = 100 MAXSTEPHALVING = 5 PCONVERGE = 1E-006(ABSOLUTE) SINGULAR = 1E-012 ANALYSISTYPE = 3(WALD) CILEVEL = 95 CITYPE = WALD LIKELIHOOD = FULL

/MISSING CLASSMISSING = EXCLUDE

/PRINT SOLUTION.

!DOEND

!ENDDEFINE.

* 7. Change the number of loops below, from X to t-1.

!SimReg NumLoops = X.

* 8. Save the lower 95% confidence limits of the intercept from each regression analysis.

* These represents the level of the influenza activity and are compared with the predetermined threshold.

* If the level is above the threshold, an alarm is raised, which means that the winter influenza season (or pandemic) has started; and if the level is below the threshold, no alarm is raised.

Prediction Module – Peak Timing Prediction

The analyses and coding of this module were performed in IBM SPSS Statistics. The code was written using the SPSS Syntax.

* Program:

* 1. Paste the data/time series into IBM SPSS Statistics, including the variables telenursing cases, time and the weekday effects.

* 2. The dependent variable is the telenursing cases adjusted for weekday effects.

* 3. The independent variable is the time, ranging from 1 to x, depending on the length of the series.

* 4. First the telenursing cases are adjusted for weekday effects.

COMPUTE Y_A = Telenursing/Weekdayseffect.

EXECUTE.

* 5. Run the initial program below for the first 7-day period.

USE ALL.

COMPUTE filter_$ = (Time>0&Time<8).

VARIABLE LABELS filter_$ 'Time>0&Time<8 (FILTER)'.

VALUE LABELS filter_$ 0 'Not Selected' 1 'Selected'.

FORMATS filter_$ (f1.0).

FILTER BY filter_$.

EXECUTE.

* Generalized Linear Models.

GENLIN Y_A WITH Time

/MODEL Time INTERCEPT = YES

DISTRIBUTION = NORMAL LINK = IDENTITY

/CRITERIA SCALE = MLE COVB = MODEL PCONVERGE = 1E-006(ABSOLUTE) SINGULAR = 1E-012 ANALYSISTYPE = 3(WALD) CILEVEL = 95 CITYPE = WALD LIKELIHOOD = FULL

/MISSING CLASSMISSING = EXCLUDE

/PRINT SOLUTION.

* 6. Run the second program below for the second 7 day period, where first the 7-day period is moved one step by calculating the new variable Time2 = Time-1.

COMPUTE Time2 = Time-1.

EXECUTE.

USE ALL.

COMPUTE filter_$ = (Time2>0&Time2<8).

VARIABLE LABELS filter_$ 'Time2>0&Time2<8 (FILTER)'.

VALUE LABELS filter_$ 0 'Not Selected' 1 'Selected'.

FORMATS filter_$ (f1.0).

FILTER BY filter_$.

EXECUTE.

* Generalized Linear Models.

GENLIN Y_A WITH Time2

/MODEL Time2 INTERCEPT = YES

DISTRIBUTION = NORMAL LINK = IDENTITY

/CRITERIA SCALE = MLE COVB = MODEL PCONVERGE = 1E-006(ABSOLUTE) SINGULAR = 1E-012 ANALYSISTYPE = 3(WALD) CILEVEL = 95 CITYPE = WALD LIKELIHOOD = FULL

/MISSING CLASSMISSING = EXCLUDE

/PRINT SOLUTION.

* 7. Run the program below t-9 times, where t is the length of the time series (in days) of interest and the number 9 covers the 2 programs already run above and the 7 days covered in the regression analysis of the (seven-day) period.

DEFINE !SimReg (NumLoops = !CMDEND)

!DO !i = 1 !TO !NumLoops

COMPUTE Time2 = Time2–1.

EXECUTE.

USE ALL.

COMPUTE filter_$ = (Time2>0&Time2<8).

VARIABLE LABELS filter_$ 'Time2>0&Time2<8 (FILTER)'.

VALUE LABELS filter_$ 0 'Not Selected' 1 'Selected'.

FORMATS filter_$ (f1.0).

FILTER BY filter_$.

EXECUTE.

*Generalized Linear Models.

GENLIN Y_A WITH Time2

/MODEL Time2 INTERCEPT = YES

DISTRIBUTION = NORMAL LINK = IDENTITY

/CRITERIA SCALE = MLE COVB = MODEL PCONVERGE = 1E-006(ABSOLUTE) SINGULAR = 1E-012 ANALYSISTYPE = 3(WALD) CILEVEL = 95 CITYPE = WALD LIKELIHOOD = FULL

/MISSING CLASSMISSING = EXCLUDE

/PRINT SOLUTION.

!DOEND

!ENDDEFINE.

* 8. Change the number of loops below, from X to t-9.

!SimReg NumLoops = X.

* 9. Save the regression coefficient for the variable time as well as the associated p-value from each regression analysis.

* Find the timing of the peak by following the description of this module provided in Online Technical Appendix 1 (https://wwwnc.cdc.gov/EID/article/24/10/17-1940-Techapp1.pdf).

Prediction Module – Peak Intensity Prediction

The analyses this module were performed in Microsoft Excel.

Program:

1. Paste the following variables in a worksheet in Microsoft Excel:

• The date in column A.

• The time *t* (day number) in column B, where *t* goes from 1 to *m* (representing the day of the peak timing). The starting day of the series, i.e., day 1, should be a couple of weeks before the epidemic begins.

• The number of known ILI-cases y_j per day in column C, where j goes from 1 to the last known observation, i.e., being the day when the peak timing prediction is made (between *m*-6 and *m*-11, see last paragraph of "Peak Timing Prediction" in Online Technical Appendix 1).

• The number of the weekday (1 = Monday, 2 = Tuesday, …7 = Sunday) in column D

• The weekday effects *w* in column E.

2. Paste/create the following parameters in the same worksheet:

• The timing of the peak *m* in cell K1 (assumed to be known, estimated in the peak timing prediction module).

• The total number of ILI-cases of the whole epidemic *T* in cell K2. *T* is unknown, but an appropriate starting value must be selected, for instance 100.

• The spread in time *s* in cell K3. *s* is also unknown, but an appropriate starting value must be selected also here, for instance 8.

• In cell K4, calculate the sum of the values in column J, representing the sum of the logarithmic likelihood (see below how this sum is calculated).

3. Create the following variables in the same worksheet:

• The probability density function of a Normal distribution (t:m,s) in column F for days 1 to *m*, by applying the Excel function *NORMDIST(x;mean;standard_dev;cumulative)*, in our case being *NORMDIST(t;m;s;FALSE)*, and more specifically

NORMDIST(column B; cell K1; cell K3; FALSE).

• In column G, multiply the values in column F with the parameter *T*, i.e., column F*cell K2, for days 1 to *m*.

• In column H, calculate the expected values E[Y] for days 1 to *m*, by multiplying the weekday effects *w* from column E to column G for each value/row, i.e., column G*column E.

• In column I, calculate the probability p(Y = y) for days 1 to the last known day, under the assumption that $Y_t \sim Poisson\ (T \times w \times f\ (t{:}\ m, s))$, and using the expected values $E[Y_t] = (T \times w \times f\ (t{:}\ m, s))$ from column H, by applying the Excel function *POISSON.DIST(x;mean;cumulative)*, in our case being *POISSON.DIST(y; column H, FALSE)*.

• In column J, the logarithmic values of column I are calculated (i.e., *LOG(column I)*).

4. Maximizing the likelihood:

• Start *Solver* in Excel. (Since Solver is not activated per default in Excel, it needs to be added first. To do so, click the *Microsoft Office Button* and then click *Excel Options*. Thereafter click *Add-ins* and the in the *Manage* box, select *Excel Add-ins* and click *Go*. In the *Add-Ins available* box, select the *Solver Add-in* check box and the click *OK*.)

• In *Set Objective*, choose cell K4 (being the value of interest) and choose to maximize it (*To: MAX*).

• In *By Changing Variable Cells*, choose cell K2 and K3. Note that appropriate starting values of K2 and K3 must be inserted manually first (see example of starting values above).

• Click the box in front of *Make Unconstrained Variables Non-Negative*.

• Run the solver by clicking on *Solve*.

• *Solver* will now estimate the values in K2 (the total number of ILI-cases of the whole epidemic) and K3 (The spread in time *s*) in such way so that cell K4 (the sum of the logarithmic likelihood) is maximized.

5. The predicted peak intensity can now be read out on day *m* (the row of the peak timing day) in column H.
